# Genetic modulation of the interleukin 6 (IL-6) system in patients with advanced gastric cancer: a background for an alternative target therapy

**DOI:** 10.1186/1471-2407-14-357

**Published:** 2014-05-22

**Authors:** Annamaria Ruzzo, Vincenzo Catalano, Emanuele Canestrari, Elisa Giacomini, Daniele Santini, Giuseppe Tonini, Bruno Vincenzi, Giammaria Fiorentini, Mauro Magnani, Francesco Graziano

**Affiliations:** 1 Department of Biomolecular Sciences, University of Urbino, Urbino, Italy; 2 Division of Medical Oncology, Azienda Ospedaliera “Ospedali Riuniti Marche Nord”, Pesaro, Italy; 3 Center for Pharmaceutical Biotechnology, University of Illinois at Chicago, Chicago, USA; 4 Division of Medical Oncology, University Campus Biomedico, Rome, Italy

**Keywords:** Interleukin 6, Gastric cancer, Survival, Prognosis, Chemotherapy, Polymorphism

## Abstract

**Abstracts:**

**Background**

IL-6 triggers oncogenic/angiogenic signals and the cytokine-dependent pro-cachexia cascade. The prognostic role of the functional *IL-6* (promoter) rs1800795 and the *IL-6R* (receptor) rs8192284 single nucleotide polymorphisms (SNP) was studied in patients with advanced gastric cancer treated with palliative chemotherapy.

**Methods**

One-hundred-sixty-one patients were genotyped for rs1800795 and rs8192284 SNPs using polymerase chain reaction based restriction fragment length polymorphism (PCR-RFLP) analysis assay. These results were studied for association with overall survival (OS).

**Results**

In 161 assessable patients, frequencies of rs1800795 G/G, G/C and C/C genotypes were 46%, 42% and 12%, respectively. Frequencies of rs8192284 A/A, A/C and C/C genotypes were 36%, 45% and 19%, respectively. Carriers of the rs1800795 G/G and rs8192284 C/C genotypes showed the worst OS. In the multivariate model, rs1800795 G/G (1.69 hazard ratio; 95% confidence interval 1.18-2.42), and rs8192284 C/C (1.78 hazard ratio; 95% confidence interval 1.12-2.83) confirmed an adverse prognostic impact.

**Conclusions**

In this population, genetic variants that up-regulate the IL-6 system showed impact on OS. This findings sustain the hypothesis that anti-IL-6 compounds deserve clinical studies as novel therapeutics in the palliative treatment of cancer patients.

## Background

Interleukin-6 (IL-6) is a four-helical protein of 184 amino acids that belongs to a large family of pleiotropic cytokine involved in numerous functions [1]. On target cells, IL-6 binds to an 80 kDa IL-6 receptor (IL-6R). The complex of IL-6 and IL-6R couples with gp130 protein and triggers intracellular signaling. Whereas gp130 is expressed on all cells, IL-6R is only present on few cells in the body including hepatocytes and some leukocytes [1]. Cells not expressing IL-6R cannot respond to the cytokine, since gp130 alone has no measurable affinity for IL-6. A soluble form of IL-6R (sIL-6R) comprising the extracellular portion of the receptor can bind IL-6 with a similar affinity as the membrane bound IL-6R. The complex of IL-6 and sIL-6R can bind to gp130 on cells, which do not express the IL-6R, and which are unresponsive to IL-6 [1]. This alternative stimulation has been called trans-signaling [2]. There is evidence that IL-6 trans-signaling possess a prevalent pro-inflammatory role, whereas classic IL-6 signaling via the membrane bound IL-6R is needed for regenerative or anti-inflammatory processes [2].

Dysregulation of the IL-6/IL-6R system has been associated with the pathogenesis of several autoimmune and inflammatory diseases in humans, and anti-IL-6 monoclonal antibodies (moAbs) have been successfully developed for the medical treatment of chronic inflammatory diseases, like rheumatoid arthritis [3]. Recently, anti-IL-6 moAbs have drawn attention for their potential effects in cancer patients [4,5]. On one side, IL-6 and other pro-inflammatory cytokines are involved in the mechanisms that promote cancer cachexia [6]. Also, there is evidence that IL-6 directly induces tumor growth and spread after triggering the canonical JAK/STAT pathway, an SHP-2 driven Ras-Raf-MAPK signaling pathway and angiogenesis [4,5]. Activation of these pathways has been documented in gastric cancer in experimental models and *in vivo*[7-16].

There is compelling evidence that circulating IL-6 levels and the levels of its trans-signaling promoting receptor (sIL-6R) are genetically-driven [17]. The single nucleotide polymorphism (SNP) rs1800795 corresponding to -174G/C SNP in the *IL-*6 gene promoter showed higher transcriptional activity in gene reporter assays [18]. *In vivo*, higher IL-6 levels were determined in carriers of the common allele in studies including both healthy subjects and patients with inflammatory diseases [18]. A common non-synonymous variant in *IL-6R* (rs8192284 A-C, also rs2228145) causes an Asp358Ala amino acid substitution within the extracellular cleavage domain of the IL-6R causing proteolytic cleavage of the membrane-bound IL-6R [19]. In *in vivo* studies, 358Ala carriers showed higher concentrations of the so-called soluble IL-6 receptor (sIL-6R), which is responsible of trans-signaling [19].

In several reports, an up-regulated IL-6/IL6R system has shown a prognostic impact in patients with hematologic malignancies and with solid tumors [20]. This background and the availability of novel IL-6 targeting moAbs [5] prompted us to investigate the possible influence of rs1800795 and rs8192284 on survival of patients with advanced gastric cancer. This information, beyond addressing a novel prognostic factor, may be relevant for the planning of clinical trials with anti-IL-6 therapies in this lethal disease.

## Methods

The study population consisted of consecutive patients with locally advanced, relapsed of metastatic gastric cancer who were treated with palliative chemotherapy at three participating Institution in Central Italy. One-hundred-seventy-five patients were homogeneously treated with both first-line and second-line palliative chemotherapy between 1998 and 2006 [21]. In 161 of 175 patients (92%) germline DNA was available from stored blood samples or obtained after DNA extraction from normal mucosa from archival paraffin-embedded tissues. Data on chemotherapy, treatment outcomes, baseline characteristics with routine blood chemistries, and follow-up were fully available for the 161 assessed patients. The study approval by the main hospital research and ethics committee (Azienda Ospedaliera “Ospedali Riuniti Marche Nord”, Pesaro) was granted by those of affiliate Institutions (University of Urbino and Campus Biomedico, Rome). Patients gave their general consent for the storage of their tissues and subsenquent use for research purposes.

### Genetic analyses

Patients’ characteristics and their outcomes were unknown to investigators performing genetic analyses. Genomic DNA extraction using the QiaAmp kit (Qiagen, Valencia, CA, USA). A polymerase chain reaction (PCR)–restriction fragment length polymorphism (RFLP) technique was used for determining the *IL-6R* rs8192284 A/C and the *IL-6* rs1800795 G/C genotypes. Genome DNA (10 ng) was used as a template and PCR was carried out using the Diatheva 2×PCR Master Mix (Diatheva, Fano, Italy) with the following conditions: 95°C 10 min ; 95°C 15 sec, 60°C 30 sec, 72°C 30 sec (35 cycles). The two PCR were performed using the following primer sets: rs1800795, *forward* 5’-TTCCCCCTAGTTGTGTCTTGC-3’ and *reverse 5’-TGGGGCTGATTGGAAACCT-3’*; rs8192284 *forward* 5’-CCTCTTCCTCCTCTATCTTCAATTTT-3’ and *reverse 5’-*AATGTGGGCAGTGGTACTGAA-3’. Primer pairs were designed using the PRIMER3 program (primer3plus.com*).* The PCR products were run on a 2% agarose gel after digestion with *Nla-III (IL-6,* rs1800795 G/C*)* or *Hind-III* (IL-6R, rs8192284 A/C) restriction enzymes. The predicted band sizes for the rs1800795 G/C genotypes after *Nla-III* digestion were G/G = 75 bp; G/C = 75 bp plus 50/25 bp; C/C = 50/25 bp; the predicted band sizes for the rs8192284 A/C genotypes after *Hind-III* digestion were A/A = 73; A/C = 73 bp plus 43/30 bp; C/C = 43/30 bp. Samples with ambiguous results were analyzed by direct sequencing using ABI PRISM *310 Genetic Analyzer (*Applied Biosystems, Foster City CA).

### Statistical plan

The primary endpoint of the study was the association between genotypes and overall survival (OS), as calculated from the start of first-line palliative chemotherapy until death. Genotypes were checked for deviation from the Hardy-Weinberg equilibrium using the Pearson X^2^ test. The X^2^ test and the Fisher's exact test were used to test associations between genotypes and categorical variables describing the clinico-pathologic features of the study population. Survival curves were plotted using the Kaplan-Meier and compared using the log-rank test. The Cox proportional hazards model was used for multivariate analysis to estimate and test demographic characteristics, clinical and genetic features for their associations with OS. In this exploratory study, no formal correction for multiple comparisons was adopted. However, all the following variables were included in multivariate Cox model: age, sex, ECOG performance status, weight loss (>5% in the four weeks before starting chemotherapy), anemia, albumin level, CEA level, tumor grading, histologic subtype according to Lauren’s classification, tumor location, liver involvement, presence of peritoneal carcinomatosis, number of metastatic sites and response to first-line chemotherapy. Assuming a 20% lowest frequency for an unfavorable genotype, 157 events would allow to detect an Hazard Ratio (HR) of 1.75 associated with this group (80% power and 5% type I error for a two-tailed test). All results were considered significant at two-sided *p* < .05 value. All analyses were performed by using the MedCalc software version 11.1 (MedCalc Software, Ostend, Belgium).

## Results

### Characteristics of patients and genotyping

One-hundred-sixty-one patients were analyzed. All of them received first and second-line chemotherapy and died after gastric cancer progression. First-line chemotherapy was oxaliplatin or cisplatin plus a fluoropyrimidne in 150 patients, or bolus/infusional 5-Fluorouracil in 11 patients. Second-line chemotherapy was 5-Fluorouracil coupled with cisplatin or oxaliplatin in 48 patients, with CPT-11 in 45 patients, with anthracycline in 33 patients, with paclitaxel or docetaxel in 25 patients, with VP-16 in 10 patients. Median survival time in the whole group was 9.4 months (range 0.4-34 months).

Carriers of the rs1800795 G/G, G/C and C/C genotypes were 74 (46%), 68 (42%) and 19 (12%), respectively. Carriers of the rs8192284 A/A, A/C and C/C genotypes were 58 (36%), 73 (45%) and 30 (19%), respectively. These frequencies did not show deviation from Hardy-Weinberg equilibrium and they are comparable with frequencies commonly observed in Caucasian populations.

Details of the characteristics of enrolled patients together with their distribution according to rs1800795 and rs8192284 genotypes are shown in Table 1. No significant association was observed except for liver involvement and rs8192284 genotypes. In particular, rs8192284 C/C carriers were prevalent in patients with liver metastases, while rs8192284 A/A carriers were prevalent in patients without liver metastases (Table 1).

**Table 1:** Characteristics of the 161 patients and distribution according to genotypes

	**Patients**		** rs1800795**				** rs8192284**		
	**no. (%)**	***C/C***	***C/G***	***G/G***		***A/A***	***A/C***	***C/C***	
			**No (%)**		**p**		**No (%)**		**p**
**Gender**					0.4				0.8
Male	109 (68)	15 (14)	47 (43)	47 (43)		38 (35)	50 (46)	21 (13)	
Female	52 (32)	4 (8)	21 (40)	27 (52)		20 (39)	23 (44)	9 (17)	
**Age (years)**					0.06				0.1
> 75	43 (27)	9 (22)	17 (39)	17 (39)		20 (47)	14 (33)	9 (20)	
< 75	118 (73)	10 (8)	51 (43)	57 (48)		38 (32)	59 (50)	21 (18)	
**ECOG PS**					0.1				0.7
0	88 (55)	10 (11)	43 (49)	35 (40)		31.(35)	42 (48)	15 (17)	
1	73 (45)	9 (12)	25 (34)	39 (54)		27 (37)	31 (43)	15 (20)	
**Weight loss**					0.8				0.3
< 5%	106.(66)	13 (12)	46 (44)	47 (44)		37 (35)	52 (49)	17 (16)	
> 5%	55 (34)	6 (11)	22 (40)	27 (49)		21 (38)	21 (38)	13 (24)	
**Anemia**					0.05				0.3
Hb ≥10 gr/dl	113 (70)	11 (10)	43 (38)	59 (52)		43 (38)	52 (43)	18 (16)	
Hb < 10 gr/dl	48 (30)	8 (17)	25 (52)	15(31)		15 (31)	21 (44)	12 (25)	
**Albumin**					0.5				0.2
> 3.5 gr/dl	89 (55)	11 (3)	34 (38)	44 (49)		30 (34)	41 (36)	18 (20)	
≤ 3.5gr/dl	72 (45)	8 (11)	34 (47)	30 (42)		28 (39)	32 (45)	12 (16)	
**CEA**					0.5				0.4
< 5 ng/ml	96 (60)	10 (10)	44 (46)	42 (44)		36 (38)	45 (47)	15 (15)	
≥ 5 ng/ml	65 (40)	9 (14)	24 (37)	32 (49)		22 (34)	28 (43)	15 (23)	
**Tumor grade**					0.1				0.07
G 1-2	78 (49)	10 (12)	27 (35)	41 (53)		29 (37)	30 (38)	19 (25)	
G 3	83 (51)	9 (11)	41 (49)	33 (40)		29 (35)	43 (52)	11 (13)	
**Histotype**					0.9				0.1
Intestinal	95 (59)	12 (13)	40 (42)	43 (45)		31 (33)	42 (44)	22 (23)	
Diffuse	66 (41)	7 (11)	28 (42)	31 (47)		27 (41)	31 (47)	8 (12)	
**First line RR**					0.07				0.1
CR + PR	76 (47)	10 (13)	25 (33)	41 (54)		27 (36)	39 (51)	10 (13)	
SD + PD	85 (53)	9 (10)	43 (51)	33 (39)		31 (36)	34 (40)	20 (24)	
**Liver mets**					0.3				0.002
Absent	98 (60)	9 (9)	44 (45)	45 (46)		40 (41)	48 (49)	10 (10)	
Present	63 (40)	10 (16)	24 (38)	29 (46)		18 (29)	25 (40)	20 (31)	
**LA/LR**					0.7				0.6
Yes	90 (56)	10 (11)	36 (40)	44 (49)		35 (39)	40 (45)	15 (16)	
No	71 (44)	9 (13)	32 (45)	30 (42)		23 (32)	33 (36)	15 (22)	
**Peritoneal mets**					0.1				0.9
Absent	84 (52)	13 (15)	35 (42)	36 (43)		30 (36)	38 (45)	16 (19)	
Present	77 (48)	6 (8)	33 (43)	38 (49)		28 (36)	35 (46)	14 (18)	
**Metastatic sites**					0.6				0.4
1-2	112 (69)	12 (10)	50 (45)	50 (45)		44 (39)	48 (43)	20 (18)	
> 2	49 (31)	7 (14)	18 (37)	24 (49)		14 (29)	25 (51)	10 (20)	

### Survival analyses

**Figure 1:**
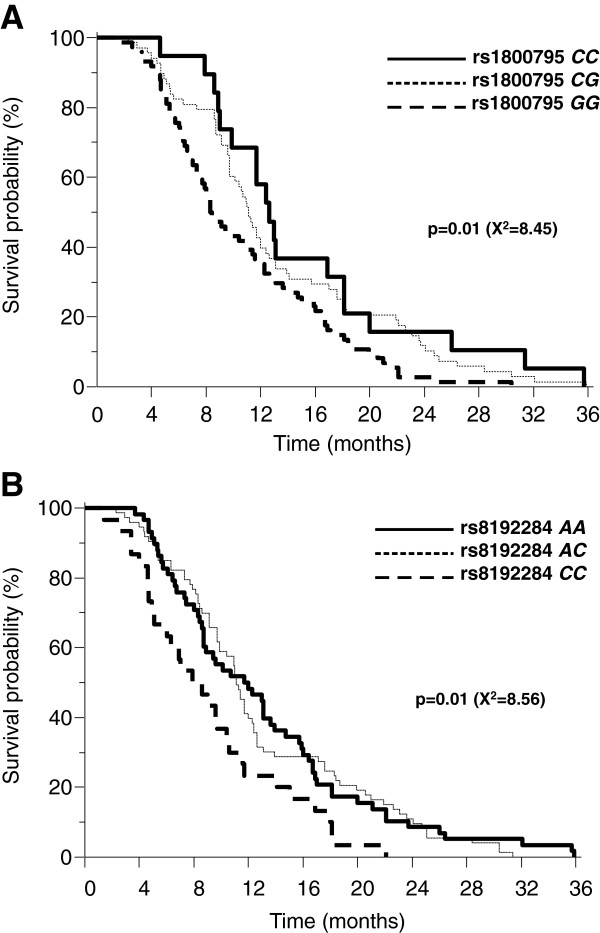
Survival analysis with distribution of patients according to rs1800795 genotypes (Panel A) and rs8192284 genotypes (Panel B).

Survival curves of carriers of the rs1800795 and rs8192284 genotypes are shown in Figure 1. In carriers of the rs1800795 G/G, G/C and C/C genotypes, median survival times were 8.4, 11 and 12.6 months, respectively (p = 0.01). In carriers of the rs8192284 A/A, A/C and C/C genotypes median survival times were 11.7, 10.1 and 8.6 months, respectively (p = 0.01).

The recessive model was adopted in the multivariate analysis with rs1800795 G/G and rs8192284 C/C defined as the risk genotypes. As shown in Table 2, harboring rs1800795 G/G (1.69 hazard ratio with 95% confidence interval 1.18-2.42), or rs8192284 C/C (1.78 hazard ratio with 95% confidence interval 1.12-2.83) confirmed an adverse impact on OS. Unfavorable survival outcomes were also significantly associated with poor performance status, lack of tumor response to first-line chemotherapy, >2 metastatic sites and the presence of peritoneal carcinomatosis.

**Table 2:** Results of the multivariate cox proportional hazards model

	**Overall survival**	
	**HR (95% CI)**	**p value**
**Gender**		
Male v. female	0.99 (0.68-1.43)	0.9
**Age (years)**		
≥ 75 v. < 75	1.11 (0.75-1.63)	0.6
**ECOG PS**		
1 vs. 2	2.04 (1.38-3.01)	0.0003
**Weight loss**		
≥5% vs. <5%	1.01 (0.59-1.35)	0.6
**Anemia**		
Hb <10 gr/dl vs. ≥10gr/dl	1.10 (0.75-1.62)	06
**Albumin**		
≤3.5 vs. >3.5 gr/dl	1.01 (0.71-1.45)	0.9
**CEA level**		
≥ 5 ng/ml vs. < 5 ng/ml	1.33 (0.91-1-94)	0.1
**Tumor grading**		
G3 vs. G1-2	0.93 (0.67-1.28)	0.6
**Histotype**		
Diffuse vs. intestinal	1.03 (0.69-1.52)	0.8
**First line response rate**		
PD + SD vs. CR + PR	1.76 (1.21-2.54)	0.002
**Liver metastasis**		
Present vs. absent	1.33 (0.89-1.98)	0.1
**LA/LR**		
Yes vs. no	0.75 (0.51-1.09)	0.1
**Peritoneal metastasis**		
Present vs. absent	1.50 (1.02-2.21)	0.03
**Number of metastatic sites**		
>2 vs. 1-2	1.90 (1.22-2.96)	0.004
**rs1800795 genotypes**		
*G/G* vs. other	1.69 (1.88-2.42)	0.003
**rs8192284 genotypes**		
*C/C* vs. other	1.78 (1.12-2.83)	0.01

**Figure 2:**
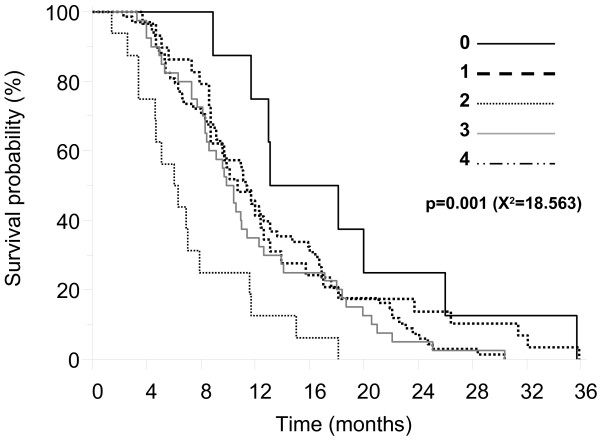
Exploratory survival analysis with classification of patients in five groups according to the number of risk alleles as defined after the primary survival analysis.

An additional explorative survival analysis was addressed to the distribution of the rs1800795 G and rs8192284 C risk alleles. There were 16 patients (10%) who were carriers of both unfavorable rs1800795 G/G and rs8192284 C/C genotypes (4 risk alleles group). Eight patients (5%) with rs1800795 C/C and rs8192284 A/A genotypes were classified without risk alleles (0 risk allele group). Twenty-nine (18%), 68 (42%) and 40 (25%) patients were grouped as carriers of 1 risk allele, 2 risk alleles, or 3 risk alleles. As shown in Figure 2, patients with 4 risk alleles showed the worst OS.

## Discussion

Clinical studies have demonstrated that increased serum IL-6 concentrations are associated with advanced tumor stages and short survival in patients with solid neoplasms [20]. IL-6 is a potent pleyotropic cytokine that may enhance a pro-inflammatory status and promote mechanisms leading to cancer cachexia in the host [1]. Also, IL-6 directly induces tumor growth and spread after triggering the canonical JAK/STAT pathway, as well as the SHP-2 driven Ras-Raf-MAPK signaling pathway and tumor angiogenesis [2-5]. Because of the restricted expression of the membrane-bound IL-6 receptor, lymphocytes and hepatocytes are the main IL-6 target cells. This pattern of receptor expression should limit the amount of cells that can respond to IL-6. However, the expression of the membrane-bound IL-6R may increase in cancer cells and alternative mechanisms may induce detrimental activation of the IL-6 system [22]. In fact, shedding of the membrane bound form into the local microenvironment, with production of the soluble form of the IL-6 receptor (sIL-6R) triggers trans-signalling, which in turn greatly increases the range of cells that can respond to IL-6 [22]. Some data indicate that sIL-6R may also act as an “orphan” molecule without complexing with IL-6 and gp130 [2]. However, the main effects of the sIL-6R seem to be agonistic with activation of trans-signaling in the presence of IL-6 [23].

There is evidence that the level of activity of IL-6 and its receptor are regulated by functional polymorphisms in the corresponding genes [17]. The common allele of a SNP in *IL-*6 promoter (rs1800795 G > C) enhances serum concentrations of IL-6 [18], while the minor allele in *IL6R* (rs8192284 A > C) is a strong inducer of the soluble form of the IL-6 receptor (sIL-6R) [19]. The minor *IL6R* allele also causes an increase in IL-6 circulating levels, but it seems an indirect effect resulting from reduced IL-6 clearance through membrane-bound IL-6R [19]. Carriers of genetic variants that up-regulate IL-6 and sIL-6R secretion may represent sub-groups of patients with a host-related feature that favors tumor growth, metastatic spread and cancer cachexia. Notably, we found that the common G allele of the *IL-*6 promoter variant (rs1800795) showed association with poor survival of patients with advanced gastric cancer treated with palliative chemotherapy. The minor *IL-6R* C allele (rs8192284) showed a weaker prognostic role than the *IL-*6 promoter variant. However, in support of a “dynamic” modulation of the IL-6/sIL-6R system, we observed a possible additive effect with worst survival outcomes in the presence of both *IL-6* and *IL-6R* unfavorable genotypes (Figure 2).

The different distribution of patients with and without liver metastasis according to the *sIL-6R* genotypes would also support the role of the IL-6/IL-6R system in the acquisition of a specific pattern of metastatic spread [24]. In experimental and *in vivo* models, IL-6 increases the metastatic potential of circulating tumor cells and modulates tissue homeostasis in a target organ of metastasis such as the liver [25]. Also, sIL-6R-mediated trans-signaling displays pro-invasive and pro-metastatic signals [1,2]. It is maximized in rs8192284 *IL-6R* minor allele carriers and it is likely to promote hematogenous spread, causing a specific pattern of metastatic disease [26].

The common *G* allele of the rs1800795 *IL-*6 promoter variant showed association with unfavorable survival outcomes of patients with ovarian cancer [27], breast cancer [28,29], neuroblastoma [30] and hematologic malignancies [31]. To the best of our knowledge, there is only one published study reporting the results of a prognostic analysis of *IL-6* polymorphisms in gastric cancer patients [32]. Liao et al [32] showed a significant association between high IL-6 circulating levels and poor survival of stage II-III, surgically resected patients, but the rs1800796 *IL-6* variant did not show prognostic role. Notably, they could not investigate the *IL-*6 rs1800795 because of the rarity of the variant allele in Asiatic populations [32], while the functional effects of the *IL-*6 rs1800796 are less extensively studied compared with the *IL-*6 rs1800795.

Less information is available on the clinical impact of the rs8192284 *IL-6R* genetic variant. In multiple myeloma patients the minor rs8192284 C allele showed association with lower overall survival [33], but in neuroblastoma patients it did not show prognostic role [31]. According to the physiology of trans-signaling and recent data on agonistic and antagonistic properties of sIL-6R [34], it is likely that *sIL-6R* and *IL-6R* genetic variants may display variable clinical effects depending on tumor type, tumor stage, concomitant treatments, host related features involving the immune system and the fine tuning of other cytokines.

Clinical data on the activation of the IL-6/IL-6R system should be considered beyond the possible prognostic role. In fact, the effects of IL-6/IL-6R may contribute to explain different sensitivity and clinical outcomes of patients treated with novel target therapies. At the same time, IL-6/IL-6R analyses could offer the opportunity of developing an alternative therapeutic strategy. In patients with metastatic renal cell cancer, high IL-6 serum levels were predictive of improved progression-free survival from the multi-kinase inhibitor Pazopanib compared with placebo [35]. In experimental models, IL-6 showed induction of cancer stem cells and epithelial-mesenchimal transition phenotype, which are possible condition for resistance to the anti-HER-2 compounds trastuzumab and lapatinib [36,37]. High IL-6 levels showed association with toxicity from Vorinostat in prostate cancer patients [38]. Anti-IL-6 molecules may counteract this, and other detrimental effects enhanced by the up-regulation of the IL-6 system. Tocilizumab, Sirukumab and Siltuximab are three MoAbs currently under investigations in clinical trials in cancer patients [5]. Ando et al [39] and Hirata et al [40] in recently published case reports, described the favorable effects on cancer cachexia and disease-related symptoms of Toclizumab in an heavily pre-treated cancer patients. Preliminary data from Phase I-II studies of anti-IL-6 in patients with multiple myeloma, castration-resistant prostate cancer and other solid tumors indicate the possible development of anti-IL-6 in cancer patients [41-43].

## Conclusion

Limitations of this study are its retrospective nature and the lack of a concomitant analysis of the cytokines circulating levels. Therefore, additional studies are needed for confirming the prognostic role of IL-6 analyses, and for corroborating the hypothesis that subjects with elevated baseline IL-6 levels and/or an IL-6 enhancing genetic profile may represent the target population for evaluating the effects of the anti-IL-6 MoAbs in cancer patients.

## Abbreviations

HR: Hazard ratio; CI: Confidence interval; ECOG PS: Eastern Cooperative Oncology Group Performance Status; RR: Response rate; CR: Complete response; PR: Partial response; SD: Stable disease; PD: Progressive disease; Mets: metastasis; LA/LR: Locally advanced/local relapse.

## Competing interests

The authors declared that they have no competing interests.

## Authors’ contributions

AR, EC, EG, MM and FG conceived and designed the experiments. VC, DS, GT, BV, GF and FG provided the samples. AR, EC, EG, MM and FG analyzed and interpreted the data. AR, EG, MM and FG contributed in the writing of the manuscript. All authors have read and approved the final manuscript.

## Pre-publication history

The pre-publication history for this paper can be accessed here:

http://www.biomedcentral.com/1471-2407/14/357/prepub
